# Louis Pasteur, a child of the Jura, a man for the world

**DOI:** 10.1093/femsre/fuae010

**Published:** 2024-04-27

**Authors:** Daniel Raichvarg, Tomasz Jagielski

**Affiliations:** University of Burgundy/EPCC Terre de Louis Pasteur, Esplanade Erasme, 21000 Dijon, France; Department of Medical Microbiology, Institute of Microbiology, Faculty of Biology, University of Warsaw, I. Miecznikowa 1, 02-096 Warsaw, Poland

**Keywords:** Louis Pasteur, Pasteur Institute, biography, infectious diseases, microbiology

## Abstract

How did Louis Pasteur, born in a small town in the Jura—Dole, still little known to the world today, become a man of global recognition and fame? The answer to this question is guided by two pivotal considerations. First is Pasteur's relationship to the representation of reality. This relationship was seeded and steadily developed since his juvenile years through practicing different forms of artistic expression, the most famous of which were subtle pastels portraying Pasteur's parents and neighbors. This genuine attraction towards art gradually became «scientificized» at the same time, when new means of reproducing the reality were invented, such as photography. The second consideration, critical to understand the phenomenon of Pasteur's celebrity, is a strong linkage of his research with nature-based agricultural production. Here again, deeply rooted in his youth and home environment, permeated with the taste of wine and the smell of tanned leather, Pasteur's interests necessitated the processes of communication, not only at the scientific level, but also on a daily life basis, with numerous «social actors» at play (ferments, silkworms etc.). Throughout his work, Pasteur had to provide himself with the means to set up these interdisciplinarity and communication. The final result was the Pasteur Institute, or rather the Pasteur Institutes and the global Pasteur network.

## Teaser

The article explains the phenomenon of Louis Pasteur, to whom the Special Issue is dedicated.

**Figure 1.**
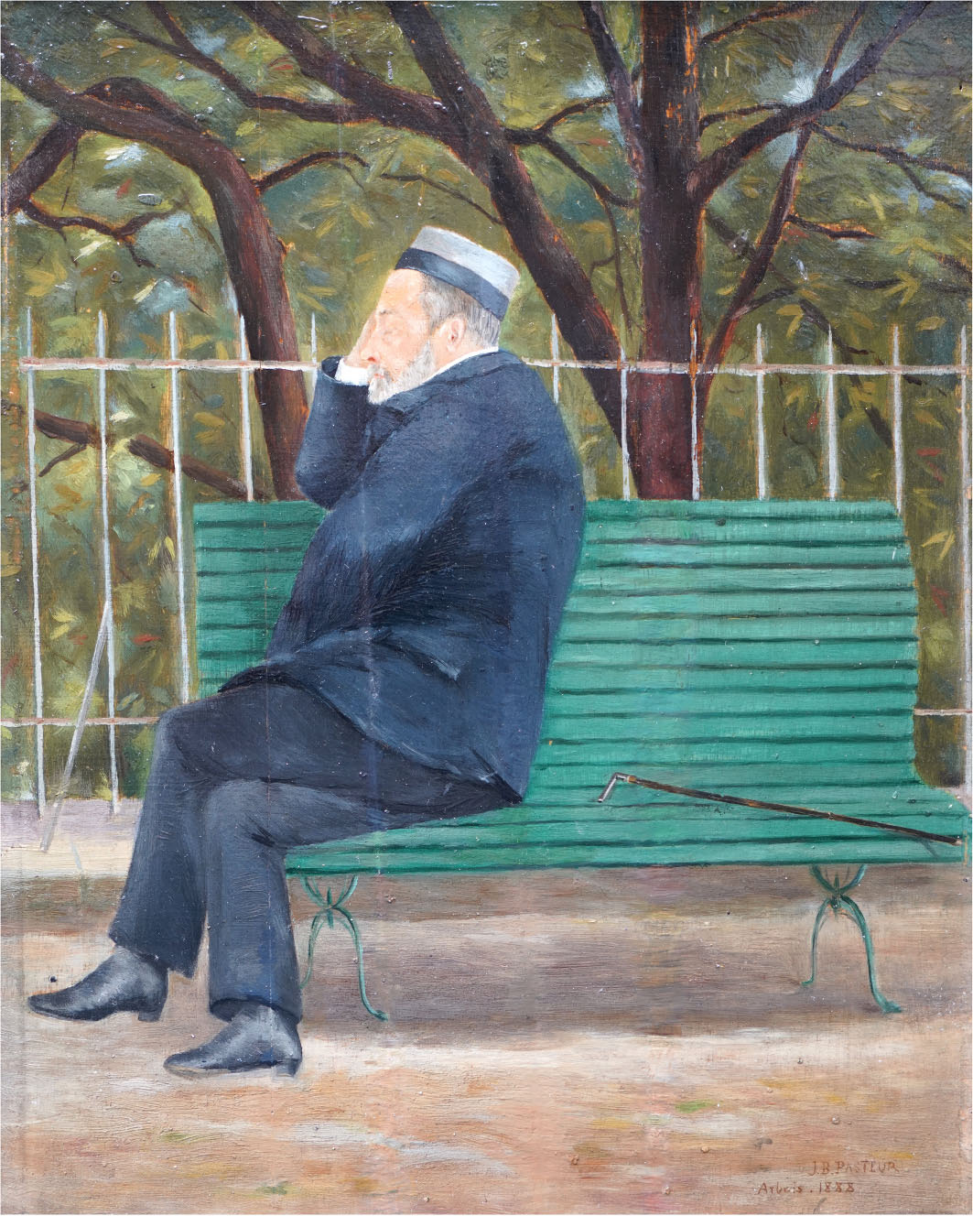
Pastuer pensive in his garden—a picture by his grandson, Jean-Baptiste Pasteur. Arbois, 1888. Private collection of Philippe Bruniaux.

France, 1822. Only 7 years have passed since the frontline canons fell silent and settled the dust of the marches of the Napoleon's *Grande Armée*. Europe is chewing the provisions of the Congress of Vienna, while France is healing the wounds after the collapse of the Empire and struggling to restore the Bourbon's monarchy. In these times, heavily replete with political, social, and economic crisis, a man comes into the world who, until his final days, will defend life and peace. A man who will make France famous, but not as Napoleon did—as a conqueror of the world and tyrant of life—but as its apologist and tireless defender. On December, 27^th^ 1822, in a small city of Dole, in the Jura district, eastern France, Louis Pasteur was born (Fig. 1). How did this son of a “poor” tanner become a man of global recognition and fame? How can the phenomenon of Pasteur's life be explained? Of paramount importance is his attachment to the representation of reality. This attachment was seeded and steadily developed since his juvenile years through practicing different forms of artistic expression, the most famous of which were subtle pastels portraying Pasteur's parents and neighbors. Although he abandoned his passion for art and took up a scientific path, even as a scientist, in order to understand a given phenomenon, he first had to study it in depth, map it in his mind, and transfer onto paper. This is clearly evidenced by almost all scientific sketches, characterized by an extraordinary perspicacity of observation, an outstanding precision of words, and exceptional accuracy of drawings. One telling example of Pasteur's genuine strategy when approaching scientific problems is provided by his works on crystallography. Trusting to his very acute and watchful eye, armed with unparalleled patience, and extremely meticulous and systematic analytical methodology, Pasteur made his first great discovery of the molecular asymmetry of tartaric acid (Vallery-Radot 1922b). This was indeed Pasteur's first capital contribution to science laying the bedrock for the theory of stereochemistry. Pasteur's investigations in crystallography led to his nomination to the Academy of Sciences in 1856. However, a young chemist from Dole lost the ballot and had to wait 5 years to be elected to the Section of Mineralogy of the Academy. At that time, he was already known for his distinguished works on fermentations and outstanding experiments refuting the theory of spontaneous generation (Debré 1994).

Another consideration, critical to understand the phenomenon of Pasteur's celebrity, is a strong linkage of his research with various manifestations of human production, especially that based on nature and the environment, such as agricultural production. This in turn stemmed from Pasteur's particular sensitivity to people's needs, respect for human ingenuity, manufacturing, and attention to social wellbeing in general. Deeply rooted in his youth and home environment, permeated with the taste of wine and the smell of tanned leather, Pasteur's interests necessitated the processes of communication, not only at the scientific level, but also on a daily life basis, with numerous «actors» at play, including brewery, wine, vinegar, and sericulture entrepreneurs, but also a newly discovered world of microbes. This multilateral «dialogue» was initiated in the mid-1850 s, when Pasteur started his works on fermentations. First he explained the cause of the lactic fermentation, relating it to the presence and growth of «living ferments» (lactic bacteria) (Vallery-Radot 1922a). Then he studied other fermentations, trying to solve the problem of the «maladies» of beer and wine. Of particular concern was the spoilage (acetification) of wines over long-distance transportation or long storage. The problem incurred heavy economic losses to the French wine industry and became a matter of state. It was Emperor Napoleon III himself who asked Pasteur for help. With great ardor Pasteur began his investigation, making a city of Arbois, in his native Jura department, a theater of the experiments. In a country laboratory, with a group of his best students from the École Normale, in full view of onlookers and amazed winegrowers, Pasteur pursued to fathom the mystery of wine «disease». He eventually found it to be due to fermenting bacteria, which, unlike yeasts, carry out non-alcoholic fermentation (e.g. acetic fermentation), whose products may spoil the wine's flavor and taste. The same conclusions Pasteur reached from studies on beer. To prevent wine and beer «disorders» Pasteur proposed a method, originally invented by Nicholas Appert, of applying heat to liquor for a short period of time, which would suppress the growth of contaminant bacteria (Vallery-Radot 1922a). The procedure, under different refinements, collectively referred to as pasteurization, is still being used for the preservation of many food products and beverages. Studies on fermentations not only brought Pasteur many patents and awards, but prepared him to disprove the theory of abiogenesis, and served as the prelude to his own germ theory of infectious diseases. Pasteur's earliest challenge in the field of infectiology were the silkworm diseases, which at the time when Pasteur began his work, had epidemic proportions and were devastating the sericulture in France, as in other European countries. It took Pasteur 5 years to disclose the origin of the diseases and propose a prophylactic strategy against them (Vallery-Radot 1926). During that time, Pasteur visited numerous silkworm nurseries, inspected countless insects, their eggs, and their varieties, and designed a number of exquisite experiments, not only proving his remarkable analytical accuracy and precision, but also his incredible insight and research intuition. The 5-year crusade against silkworm diseases reached its symbolic ending on September 12^th^, 1876 at the banquet of the international sericulture congress organized in Milan, Italy, where Pasteur offered a toast uttering these memorable words: «La science n'a pas de patrie, parce que le savoir est le patrimoine de l'humanité» [ang. Science has no country, because knowledge belongs to humanity] (Vallery-Radot 1939).

Pasteur's studies on silkworm diseases assembled in an extensive essay, referred to as «a veritable guide to whoever would study contagious disease» by Émile Roux, sowed the seeds for a revolutionary theory of germ origin of diseases, which over the next few decades sprouted into a global quest for pathogens, resulting in the discovery of the causative agents of many infectious diseases, including leprosy, tuberculosis, cholera, diphtheria or plague, and somewhat later—the development of new modalities for their prevention and treatment. Pasteur remained in the very avant-garde of that quest, having significantly contributed to the elucidation of the etiology and pathogenesis of such important diseases as fowl cholera, anthrax, swine erysipelas, and rabies. As with all his previous research, Pasteur was motivated undoubtedly by his tremendous scientific curiosity, but even more so by a strong desire to alleviate at least some of the poignant pains and miseries affecting different areas of people's activity, and through this to hoist the economy of his country and ameliorate the health and welfare of his compatriots. Thus, we return to the close connection of Pasteur's research with the historical and economic realities of his time. Pasteur deliberately directed his attention on the diseases that were ravaging the agriculture sector of his country, approaching the problem from a very practical and pragmatic perspective: how to curb the spread of the disease epidemic and how to make it preventable? The tenacious adherence to this pivotal question led Pasteur to the invention of the first live attenuated vaccines, as were those against chicken cholera or anthrax. With his studies on vaccines, Pasteur made a lasting mark in the history of science, earning a status of the forerunner of modern immunology and vaccinology. The history of vaccines provides perhaps the most compelling evidence of Pasteur's scientific greatness. It also shows how those epochal findings vaulted Pasteur to a level of global recognition, witnessing the birth of his stardom and legend. Two prominent dates could serve as important milestones and turning points in Pasteur's career, marking the beginning of his world fame. First is May 3^rd^, 1881, when Pasteur conducted, in front of a large audience, including members of the press, the first of a series of experiments demonstrating the efficacy of his anthrax vaccine (Pasteur et al. 1881). The other date is July 6^th^ 1885, when Joseph Meister, a 9-year-old boy, who had been severely bitten by a rabid dog, crossed the threshold of the Pasteur's laboratory (Vallery-Radot 1933). These two dates document two magnificent triumphs of Pasteur, which circulated around the world expeditiously, arousing frenetic enthusiasm and profound appreciation. The great success of anti-rabies vaccination entailed massive implications for the entire scientific community and for Pasteur personally. Apart from many awards and distinctions he received from across the world, he stood at the head of the new institute, established as a votive offering of the gratitude of the nation to his paramount achievements. The Pasteur Institute whose inauguration took place on November 14^th^ 1888, and was attended by hundreds of distinguished persons, including the president of the Republic of France, Sadi Carnot, was remarkably innovative for at least two reasons. First, it was a combined public and private fundraise that enabled the Institute to be set up. Second, it was a deliberately planned organizational structure of the Institute, with a leading role of two Pasteur's successors at the director position of the Institute—Émile Duclaux and Émile Roux. Pasteur himself defined three main objectives of the Institute: «Notre institut sera à la fois un dispensaire pour le traitement de la rage, un centre de recherche pour les maladies infectieuses et un centre d'enseignement» [ang. «Our institute will be a dispensary for rabies treatment, a research center for infectious diseases and a teaching center» (Pasteur 1888).

The biography of Louis Pasteur is a fascinating and dramatic tale of great ambition, bold ideas, powerful intuition, and insatiable passion. It is not a history of a prodigious child or an «isolated» genius, but rather of a brilliantly stubborn and inexhaustible Titan of work, firmly grounded in his local universe, deeply attentive to its voices and responding with abundant curiosity and exploratory zest. The history of Pasteur is a also a code of strict values, austere principles, and rigorous beliefs. Throughout his life, Pasteur persevered in obedience to this code, best exemplified by his utter devotion to his country, family, and science. So, what could best and most briefly explain the phenomenon of a child of the Jura that became a man for the world is the very essence of his life—fidelity to his precepts. Pasteur's love of the truth, unwavering commitment to science, and the utilitarian service to his nation resonating with the Promethean idea of improving the welfare of mankind, have canonized him one of the greatest minds of all time. The child of the Jura has become a man for the world, immortalized with the title of «benefactor of humanity» [fr. «bienfaiteur de l'humanité»].

Last year, the global scientific community paid tribute to Louis Pasteur, the «father of microbiology» on the occasion of the bicentenary of his birth. Celebrating this jubilee effectuated many festivities and commemorations across the world.

It should be borne in mind that such celebrations, apart from their obvious historical value, should have a strong reference to the present, convey a clear message to future generations. This was aptly expressed by Émile Borel, a mathematician and member of the French Academy of Sciences, who, in his article «The lessons of a centenary» dedicated to the 100^th^ anniversary of the birth of Louis Pasteur, published in «La Revue de Paris», wrote:

«Je crois qu'on a eu raison de donner un éclat exceptionnel au centenaire de Pasteur… Mais une telle leçon serait, malgré tout, incomplète, si, après avoir étudié le passé, nous ne regardions pas vers l'avenir. Il est peut-être né, le 27 décembre 1922, en un coin perdu de nos campagnes, dans le faubourg de l'une de nos grandes villes industrielles, un enfant qui pourrait être le Louis Pasteur du XXe siècle; que faut-il faire pour que cette force immense ne soit pas perdue? C'est le problème de l’éducation de la jeunesse qui se trouve tout d'abord posé ainsi».

[ang. «I believe that we were right to give such an exceptional radiance to Pasteur's centenary… However, such a lesson would be incomplete if, after having studied the past, we did not look to the future. On December 27^th^, 1922, in a remote part of the countryside, in the suburbs of a large industrial city, a child may have been born, who could be the Louis Pasteur of the XX^th^ century; what must be done to ensure that this tremendous strength is not lost? This is the question of educating the youth that is here raised.»] (Borel 1923).
